# Thoracic Spine CT Hidden Treasures: Lung Assessment and Extraspinal Findings in Patients with Vertebral Fractures Studied with Full FOV during Breath Hold: Technical Note

**DOI:** 10.3390/tomography8020080

**Published:** 2022-04-02

**Authors:** Antonio Manca, Gabriele Chiara, Saverio Bellizzi, Piergiorgio Valle, Silvia Nicoli, Delia Campanella, Daniele Regge

**Affiliations:** 1 Radiology Unit, Candiolo Cancer Institute, FPO-IRCCS, 10060 Torino, Italy; gabriele.chiara@ircc.it (G.C.); piergiorgio.valle@ircc.it (P.V.); delia.campanella@ircc.it (D.C.); daniele.regge@ircc.it (D.R.); 2 Independent Researcher, 1201 Geneva, Switzerland; saverio.bellizzi@gmail.com; 3 Radiology Unit, Department of Surgical Science, University of Torino, Via Genova 3, 10126 Torino, Italy; silvia.nicoli@unito.it

**Keywords:** incidental findings, thoracic vertebrae, multidetector computed tomography, pneumonia, lung neoplasms

## Abstract

Thoracic spine CTs are usually performed during free breathing and with a narrow field of view; this common practice systematically excludes the assessment of lungs and other extraspinal structures, even if these have been irradiated during the examination. At our institution we perform thoracic spine CT during breath hold with additional full FOV reconstructions; this allows us to also evaluate lungs and extraspinal pathologies in the same examination with no added costs or further radiation exposure. If this simple and costless technical change is routinely applied to thoracic spine CT many concomitant extraspinal pathologies can be ruled out, from neoplasms to pneumonia; the suggested modification also allows an early diagnosis and avoids recalling and re-irradiating the patient in case these findings are partially included in the study. This practice can be further useful during the current pandemic in order to screen any lung opacities suspicious for COVID-19.

## 1. Introduction

Computed Tomography of the thoracic spine is comparable to unenhanced CT of the thorax in terms of body coverage and radiation dose; however, the opportunity to assess lung or thoracic pathologies during spine CT scans is daily missed in most studies performed with the sole intent of assessing spinal disorders.

Thoracic spine CT, as suggested in technical protocols [1], is routinely performed during free breathing with narrow field of view (FOV) centered on vertebral bodies and multiplanar reconstructions are made with bone window and soft tissue window only.

**Figure 1:**
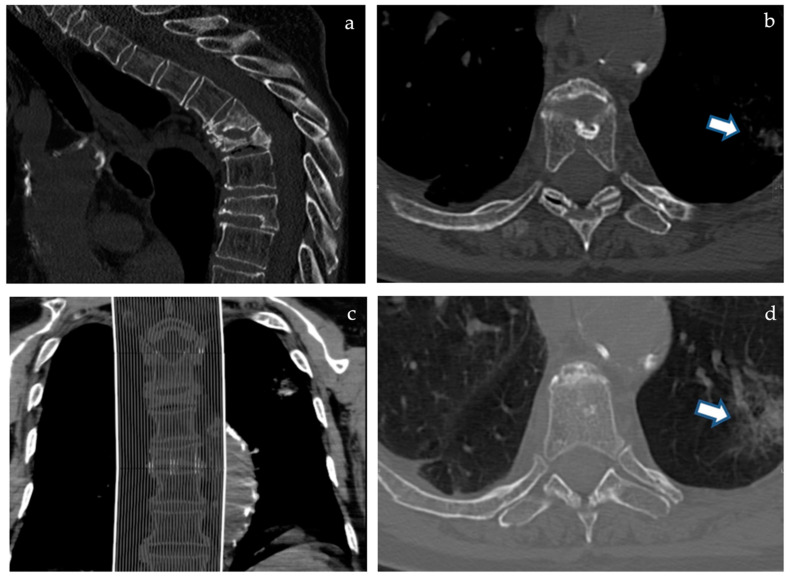
(**a**) (**above left**), (**b**) (**above right**), (**c**) (**below left**), and (**d**) (**below right**). A 77-year-old female patient with unbearable pain due to T5 and T6 spontaneous severe fractures (**a**,**c**) performed CT among a different institution due to the scarce compliance required for Magnetic Resonance. While reviewing CT imaging in our Interventional Radiology consultation office to assess the indication for vertebroplasty, a left lung opacity was noticed on “bone window” (**b**, arrow) and was confirmed and more evident when switching to “lung window” view mode (**d**, arrow).

If any pulmonary alteration is even partially included in the field of view, this may appear as a blurry opacity due to breathing artifacts and from a “through the keyhole” perspective due to narrow FOV (Figure 1b,d); whenever these incidental extraspinal findings are noticed, the patient is usually called back to undergo a standard thoracic CT, thus doubling radiation dose, costs, and discomfort of the diagnostic work-up. Given the large portion of thorax left outside the narrow FOV, many more extraspinal findings are hidden to the radiologist even if potentially detectable with the proper technical setting.

At our institution, inspired both by our expertise in vertebroplasty and the oncologic mission, whenever a thoracic spine CT is performed, we routinely acquire full FOV scans of the thorax during breath hold to evaluate lungs, ribs, and sternum as part of pre-operative imaging and work-up.

Since COVID-19 outbreak this consolidated practice has become even more useful considering that our hospital was conceived to be a “COVID-free” oncologic facility.

## 2. Materials and Methods

Our protocol for thoracic spine CT consists in a full FOV scan (sFOV) of the thorax during breath hold, as for standard thoracic imaging. Detector array and tube voltage chosen for our CT scanner (Siemens Somatom^®^ Definition Flash, Siemens Healthcare—Forcheim, Germany) are as follows: 128 × 0.6 with 120 kV and 90/100 mAs using automated exposition.

From the acquired body volume two images series are then obtained:

- Thorax series: full dFOV, 3 mm axial slices with lung window and mediastinum window. Optional: coronal MPR with lung window;
- Spine series: narrow dFOV, 2 mm axial slices and sagittal MPR images with bone and soft tissue window. Optional: Axial MPR images (oriented along true vertebral plane in case of kyphosis), Coronal MPR, and 3D Volume Rendering.

## 3. Results

**Figure 2:**
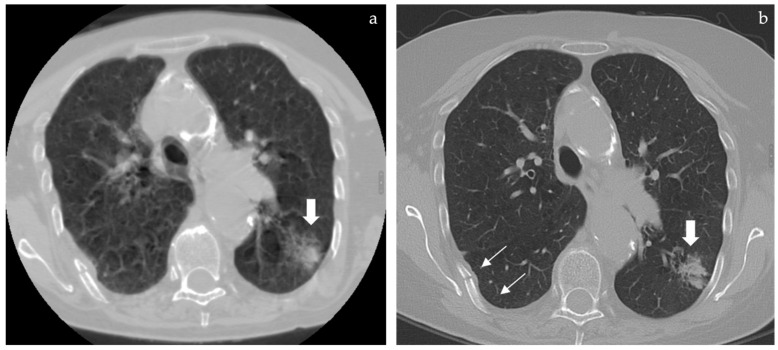
(**a**) (**left**) and (**b**) (**right**): whenever a lung opacity is spotted by chance in spine CT a full FOV reconstruction can be performed but only if RAW data are still available in the scanner. In the case (previously described in Figure 1) this allowed us to rule out further opacities but the respiratory motion artifacts prevented a proper characterization of the main lung finding that appeared as a blurry edged opacity (**a**, large arrow). The patient was called back and a standard thoracic CT was repeated to better define the lung finding that appeared as a subsolid ground glass opacity compatible with a lepidic growth adenocarcinoma (**b**, large arrow) as later confirmed by lung biopsy. In both full FOV reconstructions a rib fracture was detected (**b**, thin arrows).

Expanding the diagnostic field outside spine can be useful for various reasons:

- Assess pleural effusion that may need a pre-operative drainage both for diagnosis and to obtain an improvement of respiratory function;
- Rule out any infective opacities that may require therapy (thus delaying vertebroplasty or surgery), including COVID-19 infection that requires also prompt CT room decontamination and proper patient management;
- Assess diffuse lung diseases that may suggest a pre-operative evaluation by the pulmonologist (emphysema, bronchiectasis, and interstitial pathologies);
- Take advantage of the above listed information provided by chest CT thus avoiding to perform a pre-operative chest radiograph required by anesthesiologists;
- Rule out other causes of thoracic pain: rib and sternal fractures, subscapular elastofibroma (reported in 2.74% at CT) [2], lung or pleural pathologies with peripheral nerve involvement;

Case analysis is shown in Figure 1 and Figure 2.

## 4. Discussion

Thoracic spine CT is mainly a technological development of thoracic CT that focuses on the spine; during this evolution, a giant leap forward was done towards an in-depth evaluation of thoracic spine in 3D, different planes and with dedicated filters but at the same time a step backwards was done because with that “zooming in” somehow the “whole picture” got lost.

Sometimes technical developments need no new technologies but just a simple rethinking of what can be easily changed (just asking for a breath hold) and how much information was readily available but constantly discarded with CT scanner raw data.

In a study from Newman et al. 24% of 1239 patients were called back to repeat a CT scan of the same anatomic area within a week to have a more panoramic view because a different physician requested the additional CT scan or to clarify extraspinal findings/symptoms; one third of patients underwent thoracic spine CTs of the same body volume only to evaluate thorax (30/91 pts, 33%) [3]. The study of Newman et al. was a retrospective analysis not focused on the chest; it also included lumbar spine. The patients were studied in an emergency setting and part of the imaging integrations were requested with contrast medium. What has to be pointed out is that many CTs were repeated, even if reconstructions were still feasible, but unfortunately, the examinations were not performed during breath hold and thus, would have been repeated anyway to achieve proper chest imaging. This common practice is often due to the fact that spine CT is requested by a spine surgeon and reported by a neuroradiologist and both tend to focus on spine while a second examination is usually requested by a clinician.

Apart from the previously listed advantages, the routine inclusion of the thorax in spine CTs can represent an issue for the following reasons:

- Adds reporting time;
- More findings mean also the need to schedule a follow-up and, eventually, further diagnostic examinations (PET-CT, biopsy);
- Needs to be reimbursed;
- Need appropriate diagnostic skills in the radiologist; neuroradiologists may need a consultation of a thoracic or general radiologist to complete the report.

On the other hand, performing a full FOV thorax scan during breath hold while studying spine CT can be more cost-effective and ethically acceptable because the whole body area that has been irradiated is analyzed in the same examination and can represent a useful screening tool, both for tumoral and infective findings.

## 5. Perspective of the Patient

The purposed protocol has mainly been created in order to enhance the diagnostic content of a single CT scan while minimizing the discomfort for the patient due to the request to repeat the same examination with breath hold.

Most of our patients have vertebral fractures that need to be investigated with CT because have a complex morphology and/or the patient is too symptomatic to bare the supine position long enough to undergo an MRI and/or have contraindications to magnetic resonance (pacemaker, severe obesity, and claustrophobia). In all instances the patient can be frail, old, bed-ridden, and, thus, not easy to be transported and positioned within a gantry.

In the study from Newman et al. [3] 30 out of 91 patients (33%) who had undergone thoracic spine had to be called back to repeat the same scan in apnea in order to obtain a lung study; this percentage might become 0% just if the apnea is asked during the same examination.

On the other hand, some incidental findings are not useful in the management of the vertebral fracture and may require a follow-up or further bothersome and risky examinations. These other incidental findings are mainly adrenal incidentalomas and lung nodules. The prevalence of adrenal incidentalomas was 4.4% in the study by Bovio et al. analyzing 520 patients coming from the same geographical area who had undergone non-contrast chest CT in a screening program of lung cancer (Tic TAC study) [4]; in this population 21 adenomas, 1 myelolipoma, and 1 lung cancer metastasis were found, thus, the only malignant adrenal mass was strictly related to a lung neoplasm that may have been missed if the patient underwent just a CT of the thoracic spine.

For the management of adrenal incidentalomas the European Society of Endocrinology primarily recommends the use of non-contrast CT: if this scan is consistent with a benign adrenal mass (Hounsfield units ≤ 10) that is homogeneous and smaller than 4 cm, no further imaging is required. In all the other patients the serum creatinine value can be promptly obtained and the contrast enhanced CT can be performed within one hour: in case the incidentaloma remains undetermined, a 6–12 months follow-up with contrast-enhanced CT will be scheduled, as suggested by the same guideline [5].

Incidental lung nodules can be managed according to Fleischner society guidelines [6].

To assess if really the advantages of this protocol outweigh its disadvantages, further studies are warranted. To reach a good level of evidence, patients with thoracic fractures should be randomized and studied with thoracic spine CT alone or with chest CT in the same examination.

## 6. Conclusions

Turning every spine CT into a thoracic CT needs only to perform the examination during breath hold with additional full FOV reconstructions and this practice, if routinely applied and included in guidelines, can represent a “one-stop shop” also preventing potential medico–legal issues derived from missed or delayed diagnoses of lung and extraspinal pathologies.

Furthermore, reporting extraspinal findings in spine CTs could become a common good practice as we should be, conversely, paying attention to the spine while reading chest CTs [7].

## Data Availability

Not applicable.

## Abbreviations

FOV: field of view, sFOV: scan FOV, dFOV: display FOV, CT: computed tomography, and COVID-19: Corona virus disease 19.
